# RodZ: a key-player in cell elongation and cell division in *Escherichia coli*

**DOI:** 10.3934/microbiol.2019.4.358

**Published:** 2019-11-07

**Authors:** Risa Ago, Daisuke Shiomi

**Affiliations:** Department of Life Science, College of Science, Rikkyo University, 3-34-1 Nishi Ikebukuro, Toshima-ku, Tokyo 171-8501, Japan

**Keywords:** RodZ, MreB, FtsZ, peptidoglycan, Rod complex, divisome

## Abstract

RodZ is required for determination of cell shape in rod-shaped bacterium, such as *Escherichia coli*. RodZ is a transmembrane protein and forms a supramolecular complex called the Rod complex with other proteins, such as MreB-actin and peptidoglycan synthesis enzymes (for e.g., PBP2). Deletion of the *rodZ* gene changes the cell shape from rod to round or ovoid. Another supramolecular complex called divisome that controls cell division mainly consists of FtsZ-tubulin. MreB directly interacts with FtsZ and this interaction is critical to trigger a transition from cell elongation to cell division. Recently, we found that RodZ also directly interacts with FtsZ, and RodZ recruits MreB to the divisome. Formation of the division ring, called Z ring, is delayed if RodZ does not interact with FtsZ, indicating that RodZ might facilitate the formation of the Z ring during the cell division process. In this mini-review, we have summarized the roles of RodZ in cell elongation and cell division, especially based on our recent study.

## 1. Introduction

**Figure 1.**
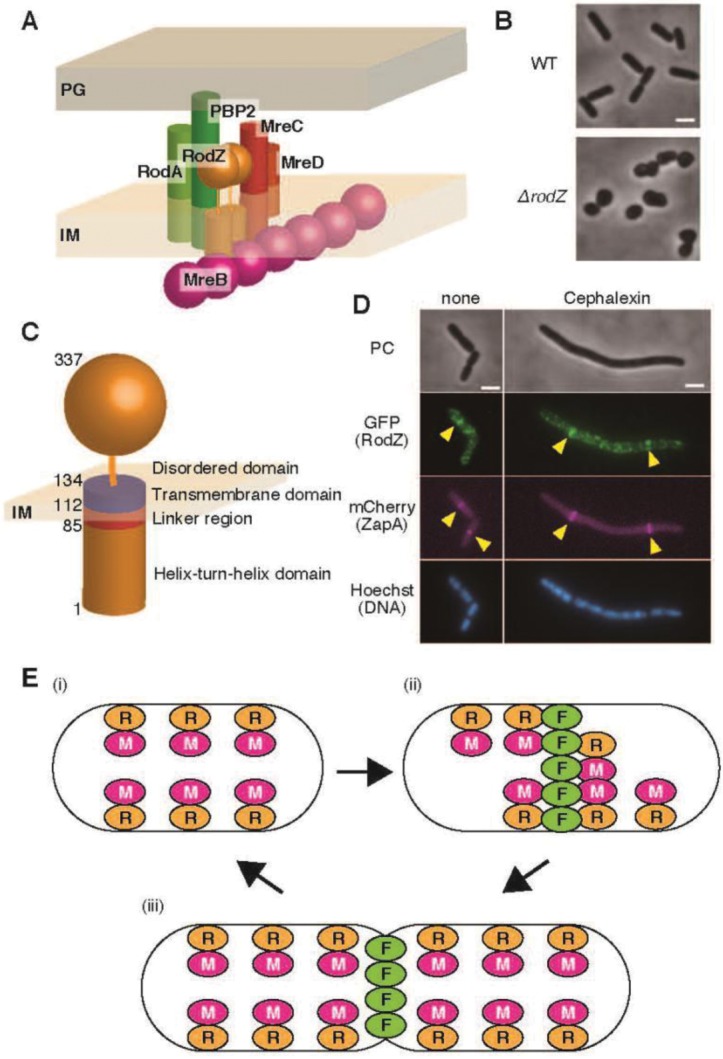
RodZ functions. (A) Schematic illustration of the Rod complex. Different components of Rod complex such as, RodZ, MreB, MreC, MreD, PBP2 and RodA are shown. Other components were not shown in this illustration. PG; peptidoglycan, IM; inner membrane. (B) Phase contrast image of WT cells and cells lacking *rodZ* (Δ*rodZ*). Scale bar is 2 µm. (C) Schematic illustration of the structure of RodZ. (D) Subcellular localization of RodZ. Phase contrast and fluorescent images of RU1124 cells producing sfGFP-RodZ and ZapA-mCherry grown in the absence and presence of cephalexin. Cells were also stained by Hoechst33342. Arrowheads indicate division site localization of RodZ and ZapA. Scale bar is 2 µm. (E) Model for functions of RodZ in cell elongation and cell division. (i) When a cell elongate, Rod complexes are formed. (ii) The structure of RodZ is changed by MreB to the suitable structure for division site and RodZ localizes to division site through direct interaction with FtsZ. (iii) A cell is contracted by divisome. R; RodZ, M; MreB, F; FtsZ.

Bacterial cells repeatedly elongate and divide to proliferate, but these two events must be precisely coordinated. Cell elongation is regulated by a supramolecular complex called Rod complex (Figure 1A), while cell division is regulated by a supramolecular complex called divisome. MreB—prokaryotic homolog of actin, and FtsZ—prokaryotic homolog of tubulin are scaffold proteins for the Rod complex and the divisome, respectively [1]. Rod complex consists of many proteins, such as MreB, a transmembrane protein RodZ, and a peptidoglycan synthesis enzyme penicillin-binding protein (PBP) 2; divisome consists of many proteins, such as FtsZ, ZipA, ZapA, and a peptidoglycan synthesis enzyme PBP3 [1],[2]. The shape of a bacterial cell is determined by a balance between elongation and division. These two events were initially thought to be independent; however, it was found that MreB localizes to the division site in an FtsZ-dependent manner [3]–[5] and PBP2 directly interacts with PBP3 [6], suggesting that the Rod complex interacts with the divisome at the division site. Furthermore, it was shown that the direct interaction between MreB and FtsZ is important for the transition from cell elongation to cell division [7]. We recently found that the transmembrane protein RodZ also localizes to the divisome and facilitates the formation of the divisome [8]. This mini-review outlines the features of RodZ, especially focusing on our recent results. Here, we describe RodZ of *Escherichia coli* unless otherwise stated.

## 2. RodZ protein

*rodZ* gene was identified independently by three research groups more than ten years ago [9]–[11]. We visually screened the KEIO knockout library (3985 strains), which is a collection of single-gene knockouts of non-essential genes in *E. coli*, to find mutant(s) that showed abnormal morphology. One of the mutants in the library (Δ*yfgA*) showed round or ovoid-shaped phenotype (Figure 1B) [9]. de Boer's group also searched for a transposon insertion mutant that required additional FtsZ for viability or better growth and showed cell-shape defects like other cell-shape mutants, such as the *mreB* mutant [12]. Consequently, they found a mutant in which transposon was inserted into the *yfgA* gene. *yfgA* was then renamed *rodZ* because the mutant *r*equired an *o*ver*d*ose of Fts*Z* for propagation in LB [10]. Furthermore, Jacobs-Wagner's group also visually screened a transposon library of *Caulobacter crescentus* and found a mutant which showed slow growth and aberrant morphology [11]. In the mutant, a transposon was inserted into *cc_0850*, which is a homolog of *rodZ*, indicating that RodZ plays a role in morphogenesis in *C. crescentus* (like in *E. coli*). It has also been shown that RodZ is required for cell shape maintenance in *Bacillus subtilis* and *Deinococcus grandis* [13],[14]. All the above reports showed that *rodZ* mutants exhibit aberrant morphology, indicating that RodZ is required for proper cell-shape maintenance. It should be noted that *rodZ* is a non-essential gene, whereas most of the other cell shape determinants, such as *mreB* act as essential genes in rich medium [12].

RodZ is a bitopic membrane protein of 337 amino acid residues (Figure 1C). It has a helix-turn-helix (HTH) domain (RodZ^1−84^) in the N-terminal cytoplasmic domain (amino acid residues 1–111). [15]. Although this domain belongs to the XRE family of transcription factors, the HTH domain does not interact with DNA, but it interacts with MreB and RodZ itself [10],[15]. Consistent with these results, it has also been reported that RodZ is not involved in segregation of chromosome in *E. coli* [10]. The HTH domain is followed by a linker region (RodZ^85−111^), also called the juxta-membrane region, which contains many positively charged residues that are not important for the key functions of RodZ, such as cell shape maintenance [16]. The functions of the transmembrane domain of RodZ (RodZ^112−133^) are currently under investigation in our lab (Ago et al., unpublished). The periplasmic domain of RodZ is largely dispensable because cells producing RodZ^1−142^ or RodZ^1−155^, which lacks most of the region, show a rod shape [9]. The structure of the periplasmic domain of RodZ from *B. subtilis* was predicted by NMR [17]. According to the prediction, the periplasmic domain is composed of disordered domains and is followed by a folded domain that contains nine ß-sheets. We showed that RodZ forms at least one dimer through its disordered domain, by *in vivo* site-specific photo-cross-linking assay [18]. Therefore, RodZ self-interacts in the cytoplasm and in the periplasm.

## 3. Role of RodZ in cell elongation

As described above, *rodZ* mutants of *E. coli*, *C. crescentus*, *B. subtilis*, and *D. grandis* showed aberrant morphology, such as round, ovoid, or shorter-rod phenotypes [9]–[11],[13],[14]. On the other hand, overproduction of RodZ resulted in elongation [9] or swelling [10] of the bacterial cells. This apparent contradiction may be explained by the amount of RodZ and MreB present in the cells, in other words, by the ratio of molecular number of RodZ and MreB. Because simultaneous overproduction of RodZ and MreB also resulted in cell elongation [10], ‘slight’ overproduction of RodZ may help MreB to assemble so that cells are able to elongate. On the other hand, ‘enormous’ overproduction of RodZ may result in perturbation of the Rod complex, causing cell elongation failure. All these results indicate that RodZ is an important factor in the maintenance of proper bacterial cell-shape. MreB is generally conserved in rod-shaped bacteria [19]. However, interestingly, RodZ is widely conserved among bacterial species, including several cocci that even lack *mreB* (such as *Streptococcus* spp. and *Staphylococcus* spp.) [11],[20]. This suggests that RodZ has a general role in cell morphogenesis, such as regulation of peptidoglycan synthesis. Consistent with this hypothesis, it was observed that RodZ in *C. crescentus* localizes at sites where peptidoglycan is synthesized [11].

Subcellular localization of RodZ has been previously examined. We originally observed the localization of green fluorescent protein (GFP)-RodZ expressed from a plasmid [9] and de Boer's lab observed GFP-RodZ expressed from chromosomal *att*HK022 site [10]. Recently, we constructed strains expressing super folder GFP (sfGFP)-RodZ or monomeric sfGFP (msfGFP) -RodZ from its native locus [8],[18]. It should be noted that RodZ fused with GFP (or its variants) is fully capable of maintaining the rod shape. These experiments showed that RodZ formed clusters or spiral-like structures in the cylindrical part of a cell (Figure 1D). This localization pattern of RodZ was reminiscent of that of MreB [21],[22], a major component of the Rod complex, suggesting that RodZ is also involved in the Rod complex. In the absence of MreB, RodZ was distributed throughout the cytoplasmic membrane, indicating that MreB directs localization of RodZ [10]. However, when cells producing GFP-RodZ were treated with A22, a drug that inhibits assembly of MreB [23],[24], GFP-RodZ still showed clusters or filament-like pattern. This suggests that MreB is not required for maintenance of RodZ clusters, but is required for their initial assembly. MreB-mCherry^SW^, in which mCherry was inserted between G228 and D229 of MreB, was expressed from its native locus and formed aberrant clusters in Δ*rodZ* cells [10]. On the other hand, GFP-MreB, which was expressed from a plasmid, still showed filamentous structures in Δ*rodZ* cells [9]. This suggests that RodZ is required for proper assembly of MreB, although MreB, when overproduced, can form clusters or filaments in the absence of RodZ. It should be noted that GFP-MreB fusion is not functional and that it was reported that the helical structures observed using YFP-MreB were artifacts in *E. coli* [25]. Therefore, the results from experiments using GFP-MreB fusion are hard to interpret. It was shown that RodZ does not affect polymer length of MreB (WT = 515 ± 15 nm, Δ*rodZ* = 509 ± 18 nm), but significantly affects total MreB polymer number per cell (WT = 11.6 ± 0.5, Δ*rodZ* = 6.6 ± 0.3) [26]. Bimolecular fluorescence complementation (BiFC) has been used to show that MreB interacts with RodZ, and that RodZ^1−142^ and RodZ^1−111^ still interact with MreB [27], supporting the above results. Interestingly, MreB polymer numbers per cell in cells producing RodZ^1−142^ or RodZ^1−111^ were decreased compared with those in WT cells [26]. These results suggest that RodZ regulates MreB polymer number directly and indirectly through other proteins.

Biochemical analyses revealed that RodZ directly interacts with MreB *in vitro* [15]. Purified RodZ^1−104^, the cytoplasmic fragment of RodZ from *Thermotoga maritima*, interacts with both monomeric and oligomeric MreB from *T. maritima*. The structure of RodZ^1−88^ from *T. maritima* was solved as a co-crystal with MreB [15]. The authors mutated the binding sites in RodZ molecule and concluded that RodZ interacts with subdomain IIA in MreB. *T. maritima* RodZ^Y53A^ and RodZ^Y57A^ (corresponding to *E. coli* RodZ^F60A^ and RodZ^Y64A^, respectively) mutants, in which mutation sites are located at the interface with subdomain IIA of MreB, decreased RodZ binding ability to MreB. In addition to BiFC, fluorescence resonance energy transfer (FRET) and bacterial two-hybrid (BACTH) assays also revealed that RodZ interacts with MreB *in vivo* [15],[28]. Furthermore, it was observed that GFP-RodZ^1−138−F60A^-RFP and sfGFP-RodZ^F60A^ did not form clusters, but were diffused [8],[15], consistent with the result that RodZ^F60A^ had decreased interaction with MreB. This suggests that MreB is responsible for localization of RodZ. It has been observed that MreB localizes to the cylindrical part of a rod-shaped cell. This localization pattern is accomplished by two factors; curvature-dependent cylindrical localization of MreB [29] and exclusion of MreB from cell poles by anionic phospholipids [30]. RodZ regulates curvature sensing and polymer numbers of MreB, although it is still controversial [26],[31],[32]. Thus, it can be said that RodZ might regulate localization of MreB. MreB is not static but highly dynamic; MreB rotates perpendicular to the long axis of a cell [33]–[35], and this rotation is not absolutely required for a rod shape because Δ*rodZ* cells producing MreB^S14A^, which does not rotate, can form a rod shape, although they are slightly fat [27]. RodZ shows the same motion as MreB and plays a role in linking between MreB and PBP2/RodA, which are peptidoglycan synthases [27]. In summary, RodZ and MreB are important to each other's clustering and localization and motion of the Rod complex.

Besides MreB, RodZ interacts with multiple proteins involved in the Rod complex. RodZ interacts with MreC, MreD, PBP2, and RodA, as shown in BACTH and Bimolecular fluorescence complementation (BiFC) assays [10],[27]. We also revealed genetic interaction between RodZ and MreB, RodA and PBP2 [36]. Growth of cells lacking *rodZ* is slower than that of WT cells. We isolated suppressor mutants that restored the growth defect of Δ*rodZ* cells [36]. These suppressors also restored rod shape, suggesting a connection between cell shape and growth rate in *E. coli*. Faster-growing *rodZ* mutants are also suppressed for the shape defect, as most of the mutants grew more rod-like than did Δ*rodZ* cells. This indicates a connection between cell shape and growth rate in *E. coli*. Whole genome sequencing of the suppressor mutants identified the mutation sites and showed that twenty suppressors had a mutation in the *mreB* gene (e.g., *mreB^A125V^*), four suppressors had a mutation in *mrdA* (encoding PBP2), two suppressors had a mutation in *mrdB* (encoding RodA), and one suppressor had a mutation in the promoter region of *zipA*. These proteins, except for ZipA, are components of the Rod complex. Therefore, this strongly suggests that RodZ is also a component of the Rod complex. The mutation sites on the three-dimensional structure of MreB were mostly mapped at the lateral contact sites of the antiparallel double filaments of MreB [36],[37]. This suggests that RodZ modulates the lateral contact of MreB filaments. We also isolated suppressor mutants of a chimeric protein in which the transmembrane domain of RodZ was replaced by another protein and found mutations in *mreC*, *mreD*, *mrdA*, *mrdB* in addition to *mreB* (Ago et al., manuscript in preparation). These suppressor mutations were mapped on protein-protein interaction surfaces, suggesting that RodZ modulates protein-protein interaction in the Rod complex in addition to the regulation of subcellular localization of MreB.

## 4. Role of RodZ in cell division

During proliferation, cells elongate and divide continuously. When cells divide, they change the sites of peptidoglycan synthesis from the cylindrical part to the division site. PBP2, an elongation-specific peptidoglycan synthesis enzyme, and PBP3, a division-specific peptidoglycan synthesis enzyme, colocalize during the early period of maturation of the division machinery [6], indicating that the Rod complex and the divisome interact with each other for transition from cell elongation to division. It was also shown that MreB localizes to the division site in an FtsZ-dependent manner [4],[5] and MreB directly interacts with FtsZ [7]. The interaction between MreB and FtsZ is required for the transition from elongation to division because cells producing MreB^D285A^, which does not interact with FtsZ, do not divide but can elongate [7]. Because RodZ directly interacts with MreB [15] and possibly modulates a property of MreB filaments [36], it is possible that RodZ also localizes to the division site. We recently found that RodZ also localized to the division site in an FtsZ-dependent manner (Figure 1D) [8]. RodZ directly interacts with FtsZ through the N-terminal cytoplasmic domain. Additionally, localization of ZapA, a component of the divisome, was delayed when RodZ did not localize to the division site. These results suggest that RodZ is not essential for cell division, but accelerates or stabilizes the divisome for efficient division. It is plausible that MreB recruits RodZ to the division site. In fact, RodZ does not localize to the division site in cells lacking *mreB*. However, unexpectedly, we also found that RodZ^F60A^, which decreases the binding ability of RodZ to MreB [15], localized to the division site. Only 4.7% of the cells in the population showed division site localization of MreB in cells producing RodZ^F60A^. Furthermore, overproduction of RodZ^F60A^ increased the proportion of cells showing localization of MreB (27.3%) to the division site. These results suggest that RodZ is required for the division site localization of MreB. Consistent with these results, MreB^A125V^ did not localize to the division site in Δ*rodZ* cells. It should be noted that Δ*rodZ* cells producing MreB^A125V^ are rod-shaped because the A125V mutation of MreB serves as a suppressor mutation of Δ*rodZ*.

As described above, cells cannot divide but can elongate if MreB does not localize to the division site [7]. However, cells producing RodZ^F60A^ are able to divide although, MreB does not localize to the division site, suggesting that the division site localization of MreB is not required for cell division [8]. We can explain this apparent contradiction as follows. Proliferation of bacterial cells is determined by a balance between peptidoglycan synthesis for elongation by the Rod complex and for division by divisome. MreB^D285A^ does not interact with FtsZ, but with RodZ and MreC, such that the MreB mutant is able to form the Rod complex. Thus, cells producing MreB^D285A^ continue to elongate. On the other hand, RodZ does not localize to the division site in Δ*mreB* cells. Interestingly, RodZ^F60A^ localizes to the division site and cells producing RodZ^F60A^ are able to elongate and divide. RodZ^F60A^ can interact with FtsZ and MreB although the interaction between RodZ^F60A^ and MreB is significantly weaker than that between WT RodZ and MreB [8]. This results in formation of a ‘weak’ Rod complex. Interaction between RodZ and MreB may be required for conformational change of RodZ to a suitable structure for division site localization. Weak interaction between MreB and RodZ^F60A^ may be sufficient for the structural change of RodZ. Therefore, the weak Rod complex can localize to the division site and promote cell division. The balance between peptidoglycan synthesis for elongation by the Rod complex and for division by the divisome may be appropriate in cells producing RodZ^F60A^. Thus, cells producing RodZ^F60A^ are able to divide, although MreB does not localize properly to the division site in the same cell. In the absence of MreB, the structure of RodZ fails to change to the suitable structure for division site localization, so that RodZ does not localize to the division site. Another interesting result in our study is that RodZ^85−337^ and RodZ^104−337^, which lack most of the cytoplasmic domain and clearly have a decreased ability to interact with FtsZ, still localized to the division site, suggesting that the periplasmic domain of RodZ interacts with the periplasmic domain of a component(s) of the divisome; PBP3 (also called FtsI) could be this component because it is a division-specific peptidoglycan synthesis enzyme and interacts with PBP2. This interaction would also be important for transition from cell elongation to division or stabilization of the divisome for cell division. Taken together, RodZ plays roles in both cell elongation and cell division (Figure 1F).

## 5. Perspective

Several reports have revealed interactions among proteins of the Rod complex and the divisome, and the biological significance of those interactions. In *E. coli*, the Rod complex and the divisome colocalize at the division site during the early stages of cell division [6]. It has been shown that MreB localizes to the division site in an FtsZ-dependent manner [4],[22] and it directly interacts with FtsZ [7]. RodZ also localizes to the division site, but the division site localization of RodZ is not essential for cell division because even Δ*rodZ* cells can divide; however, it has been shown that RodZ promotes the formation of the divisome during the early stages of cell division [8]. In *C. crescentus* also, RodZ localizes to the division site. This localization is dependent on both FtsZ and MreB, which means that MreB might be recruiting RodZ to the division site [11]. The authors showed that GFP-RodZ did not localize to the division site in cells producing MreB^Q26P^, which is a mutant that is unable to localize to the division site. However, cells producing MreB^Q26P^ had normal shape and divided properly, suggesting that RodZ and MreB are not critical for cell division in *C. crescentus*. In *C. crescentus* and *E. coli*, FtsZ directs peptidoglycan synthesis for elongation, which is called PIPS (PBP3-independent peptidoglycan synthesis) or preseptal elongation, at the division site. RodZ may be involved in PIPS. RodZ is widely conserved among various bacterial cells, including ovoid-shaped bacteria that lack *mreB*, such as *Streptococcus pneumoniae* and *Staphylococcus aureus*. In ovoid-shaped *S. pneumoniae* cells, there are two types of peptidoglycan synthesis; peripheral growth and septal growth [38]. Peripheral growth in *S. pneumoniae* may be similar to PIPS in *E. coli*. Thus, RodZ in *S. pneumoniae* may play a role in the peripheral growth because MreC and MreD, whose homologs are components of the Rod complex in *E. coli*, are required for peripheral growth [39]. On the other hand, in *S. aureus*, MreC and MreD localize to the division site, but are not critical for cell division and cell shape determination [40]. Therefore, RodZ may not be involved in the regulation of cell division in *S. aureus*. In *Chlamydia*, which has no homolog of FtsZ: an important protein for cell division in the majority of bacterial species, MreB plays a role in cell division [41] and RodZ interacts with MreB [42],[43]. Thus, RodZ in *Chlamydia* may also be important for cell division, although the underlying molecular mechanism is still unknown. All these reports suggest that biological functions of the division site localization of RodZ vary in different bacterial species. There are several reports on the roles of RodZ in other cellular processes such as regulation of post-transcription of type III secretion, biofilm formation, and spatial regulation of other proteins [16],[44]–[47]; these should be discussed in the future to understand bona fide roles of RodZ in different cellular processes, including cell division and cell elongation.
